# Global Change Could Amplify Fire Effects on Soil Greenhouse Gas Emissions

**DOI:** 10.1371/journal.pone.0020105

**Published:** 2011-06-08

**Authors:** Audrey Niboyet, Jamie R. Brown, Paul Dijkstra, Joseph C. Blankinship, Paul W. Leadley, Xavier Le Roux, Laure Barthes, Romain L. Barnard, Christopher B. Field, Bruce A. Hungate

**Affiliations:** 1 Laboratoire Ecologie, Systématique et Evolution, UMR 8079 Université Paris-Sud 11/CNRS/AgroParisTech, Université Paris-Sud 11, Orsay, France; 2 Department of Biological Sciences and Merriam-Powell Center for Environmental Research, Northern Arizona University, Flagstaff, Arizona, United States of America; 3 Laboratoire d'Ecologie Microbienne, UMR 5557 CNRS, USC 1193 INRA, Université Lyon 1, Villeurbanne, France; 4 Institute of Plant Sciences, ETH Zürich, Zürich, Switzerland; 5 Department of Global Ecology, Carnegie Institution for Science, Stanford, California, United States of America; Duke University, United States of America

## Abstract

**Background:**

Little is known about the combined impacts of global environmental changes and ecological disturbances on ecosystem functioning, even though such combined impacts might play critical roles in shaping ecosystem processes that can in turn feed back to climate change, such as soil emissions of greenhouse gases.

**Methodology/Principal Findings:**

We took advantage of an accidental, low-severity wildfire that burned part of a long-term global change experiment to investigate the interactive effects of a fire disturbance and increases in CO_2_ concentration, precipitation and nitrogen supply on soil nitrous oxide (N_2_O) emissions in a grassland ecosystem. We examined the responses of soil N_2_O emissions, as well as the responses of the two main microbial processes contributing to soil N_2_O production – nitrification and denitrification – and of their main drivers. We show that the fire disturbance greatly increased soil N_2_O emissions over a three-year period, and that elevated CO_2_ and enhanced nitrogen supply amplified fire effects on soil N_2_O emissions: emissions increased by a factor of two with fire alone and by a factor of six under the combined influence of fire, elevated CO_2_ and nitrogen. We also provide evidence that this response was caused by increased microbial denitrification, resulting from increased soil moisture and soil carbon and nitrogen availability in the burned and fertilized plots.

**Conclusions/Significance:**

Our results indicate that the combined effects of fire and global environmental changes can exceed their effects in isolation, thereby creating unexpected feedbacks to soil greenhouse gas emissions. These findings highlight the need to further explore the impacts of ecological disturbances on ecosystem functioning in the context of global change if we wish to be able to model future soil greenhouse gas emissions with greater confidence.

## Introduction

Human-caused global environmental changes, such as rising atmospheric CO_2_ concentration, climate change [1] and enhanced nitrogen (N) deposition [2]–[3] have increasingly recognized impacts on the functioning of terrestrial ecosystems [4]. However, the combined effects of these environmental changes on ecosystem processes are poorly understood [5]–[6], and understanding these combined effects remains critical for predicting ecosystems response to concurrent changes in the environment [7]. Furthermore, the effects of these environmental changes have not yet been examined in combination with natural disturbances such as fires, although such combined effects might play important roles in shaping ecosystem processes [8]–[9]. Here, we examine the response of soil emissions of nitrous oxide (N_2_O) - a potent and long-lived greenhouse gas [10] - to the interactive effects of simulated global environmental changes and fire in a Mediterranean grassland ecosystem.

Global environmental changes and fire can both enhance the release of N_2_O from soils through their effects on the two main microbial processes contributing to soil N_2_O production [11]–[12] – nitrification [13]–[14] and denitrification [15]. Rising atmospheric CO_2_ concentration has been found to stimulate soil N_2_O production [16], an effect mainly attributed to increased denitrification-associated N_2_O efflux [17]–[19]. This effect could result from enhanced root-derived soil carbon (C) providing energy for heterotrophic denitrification [17]–[18] and increased soil moisture reducing soil oxygen concentration and enhancing anaerobic denitrification [19]. Enhanced atmospheric N deposition and N fertilization have also been reported to increase soil N_2_O production [20], an effect associated with increased soil ammonium and nitrate contents leading to increased in both nitrification- and denitrification-associated N_2_O efflux [21]. Finally, fire can also stimulate the release of N_2_O from soils [20]. This effect has been attributed to increased nitrification-associated N_2_O efflux resulting from increased levels of ammonium in fire-impacted soils [22], and has been shown to persist up to several months after fire [23]–[25]. Thus, there is evidence that elevated CO_2_, enhanced N supply and fire can increase soil N_2_O production, but whether their effects will be additive, synergistic (amplifying each other) or antagonistic (counteracting each other) has not been studied.

We investigated the interactive effects of simulated global environmental changes and fire on soil N_2_O emissions as part of the Jasper Ridge Global Change Experiment (CA, USA). This field experiment, initiated in 1998, was initially designed to assess the interactive effects of four global environmental changes - elevated CO_2_, warming, increased precipitation, and enhanced N supply - at levels projected for the second half of the 21^st^ century in an annual grassland ecosystem [6], [26]–[27]. However, almost five years after the start of the experiment, an accidental, rapid and low-intensity fire burned two of the eight replicates [8]–[9]. This provided a unique opportunity to investigate the interactive effects between fire and global environmental changes. Warming, the treatment that had the weakest effects on nitrification and denitrification prior to the fire [28], was discontinued in the burned plots in order to increase the number of replicates for each treatment combining fire and other global environmental changes. Thus, the new field experiment consisted of a complete factorial design with four factors at two levels – burn (unburned vs. burned), CO_2_ (ambient vs. 680 µmol mol^−1^), precipitation (ambient vs. +50% above ambient) and N supply (ambient vs. +7 g N-Ca(NO_3_)_2_ m^−2^ yr^−1^) – and a total of 16 treatment combinations.

Here, we report the response of soil N_2_O emissions to the combined effects of fire and elevated levels of CO_2_, precipitation, and N supply during the three years following the fire disturbance at the Jasper Ridge Global Change Experiment. We also report the responses of related N cycling processes, including nitrification and denitrification, and of their main drivers to the treatments. Our objectives were (i) to assess the effects of the fire disturbance on soil N_2_O emissions and investigate the interactive effects of fire and global environmental changes on soil N_2_O emissions, (ii) to identify the mechanisms controlling the response of soil N_2_O emissions to the treatments, and (iii) to assess the duration of the response of soil N_2_O emissions to the fire disturbance.

## Results and Discussion

### Soil N_2_O emissions responses to fire and global environmental changes

Responses of soil N_2_O emissions to the interactive effects of fire and elevated levels of CO_2_, precipitation, and N supply were investigated during the three years following the fire (i.e. 9, 15, 19, 21 and 33 months after fire). Prior to analysis, we verified that, prior to the fire, soil and plant characteristics were indistinguishable between the plots that later burned and those that did not (Table S1). We also verified that, for each measurement date, soil N_2_O emissions were indistinguishable between the previously warmed burned plots and the previously not-warmed burned plots (Table S2). This ensured that any significant effects of the burn “treatment” on soil N_2_O emissions could be attributed to the fire disturbance.

**Figure 1:**
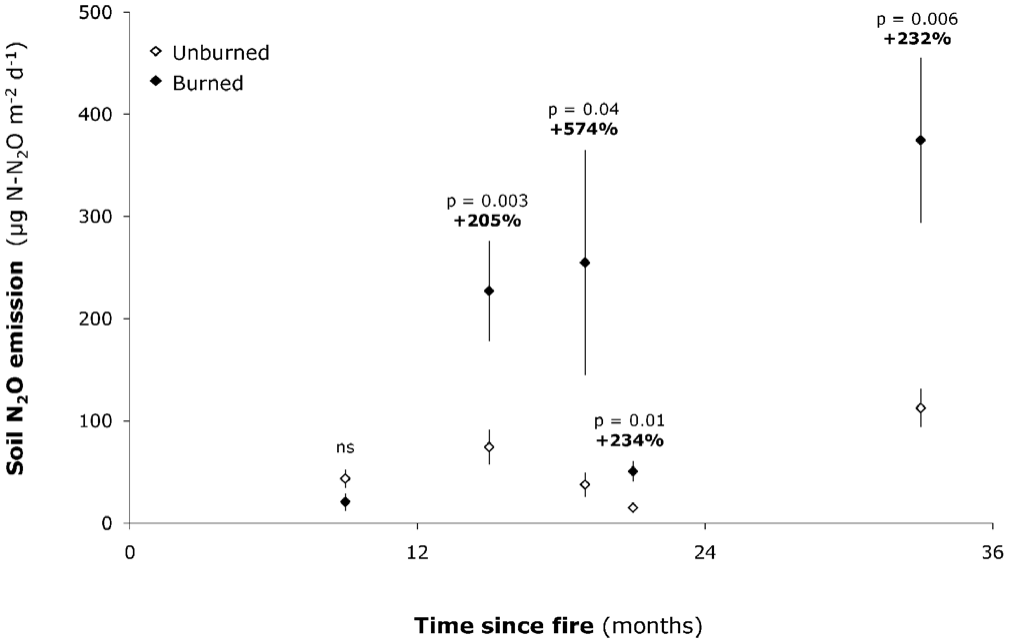
Soil N_2_O emission rates in the unburned and burned plots in relation to time since fire. For each measurement date (i.e. 9, 15, 19, 21 and 33 months after fire), soil N_2_O emission rates are grouped by burn treatments (unburned: open symbols, burned: closed symbols) and averaged across the CO_2_, precipitation and N treatments. Error bars denote standard error (n = 48 for unburned plots; n = 32 for burned plots). Results from mixed model analysis testing for a fire effect (ns: not significant) as well as relative fire effect sizes (calculated as: % effect = 100×[burned−unburned]/[unburned]) are indicated.

Fire did not significantly alter soil N_2_O emission rates at the end of the first year following the disturbance (Fig. 1). In contrast, during the second and third years after fire, we observed large increases in soil N_2_O emission rates in the burned compared to unburned plots (+205% 15 months after fire, *P* = 0.003; +574% 19 months after fire, *P* = 0.04; +234% 21 months after fire, *P* = 0.01; +232% 33 months after fire, *P* = 0.006; Fig. 1). As a result, averaged across all measurement dates, soil N_2_O production rates were substantially higher in the burned compared to unburned plots (56±30 µg N-N_2_O m^−2^ d^−1^ in the unburned plots vs. 185±145 µg N-N_2_O m^−2^ d^−1^ in the burned plots, +227%, *P* = 0.001; Tables S3 and S4). In line with other grassland studies [29], soil N_2_O emission rates showed high temporal variation at our site: the lowest soil N_2_O emission rates occurred 9 months and 21 months after fire (Fig. 1) when soil moisture was low (12% and 18%, respectively), while the highest emission rates occurred 33 months after fire (Fig. 1) when soil moisture was high (25%). Soil N_2_O emission rates also showed particularly high variation in the burned plots (Fig. 1). We therefore checked whether the response of soil N_2_O emissions to fire was driven by a response of specific burned plots. We found that, for each date, some of the burned plots showed very high soil N_2_O emission rates compared to the others, which explains the very large variance, but the burned plots showing these very high soil N_2_O emission rates differed depending on measurement date (not shown), indicating that the response of soil N_2_O emissions to fire was not driven by a specific response of some of the burned plots.

**Figure 2:**
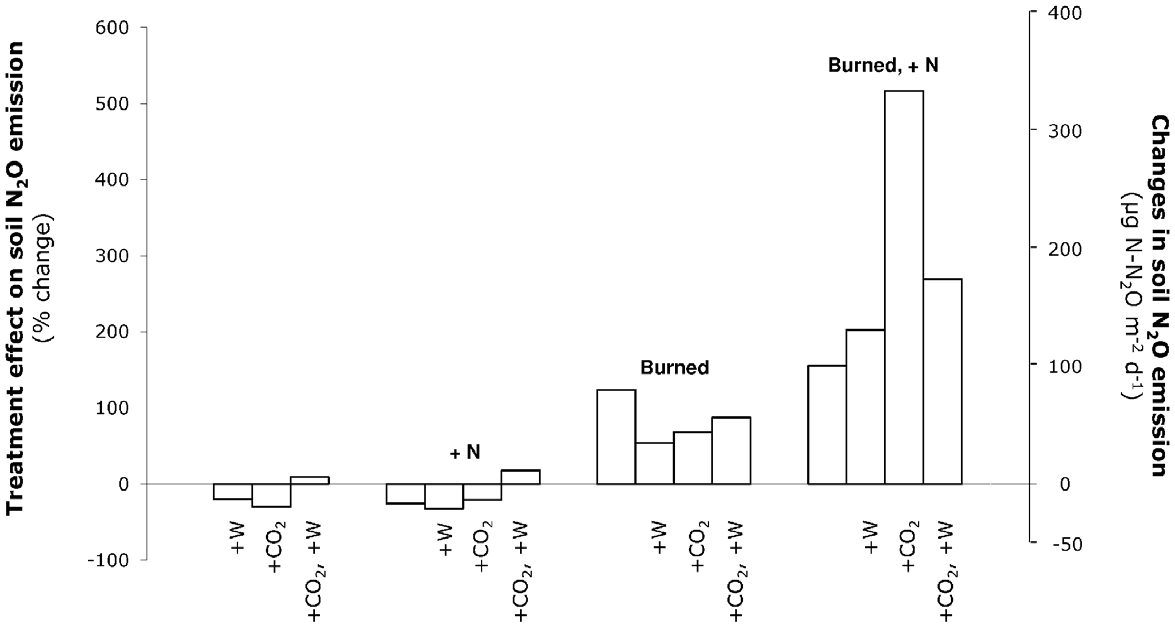
Treatment effects on soil N_2_O emission rates. Treatments are increased precipitation (+W), elevated CO_2_ (+CO_2_), enhanced N supply (+N), burn (Burned), and all their combinations. Treatment effects were calculated across the five measurement dates (i.e. 9, 15, 19, 21 and 33 months after fire) as: % effect = 100×[treatment−control]/[control]. In the control treatment, all factors are ambient. Changes in soil N_2_O emission rates in each treatment combination relative to the control treatment are also indicated. In the control treatment, soil N_2_O emission rates were 65±103 µg N-N_2_O m^−2^ d^−1^ (values indicate mean ± pooled standard error; n = 6×5 sampling dates).

Soil N_2_O emission rates exhibited a strongly non-additive response to fire and other global environmental changes (Fig. 2). Increases in soil N_2_O emission rates were larger in the burned plots when also exposed to enhanced N supply (*P* = 0.01 for the Burn×N interaction; Table S3; Fig. 2). Furthermore, the largest increases in soil N_2_O emission rates occurred in the treatment combining fire, elevated CO_2_ and enhanced N supply (*P* = 0.0007 for the Burn×CO_2_×N×Time interaction; Table S3; Fig. 2): soil N_2_O emission rates were little affected by elevated CO_2_ (−30%) or enhanced N supply (−25%) in isolation, were approximately doubled in the burned plots (+124%), and increased by 516% in the burned plots exposed to elevated CO_2_ and enhanced N supply (Fig. 2). This interactive effect was most pronounced 19 months after fire: at this time, CO_2_, N, and burn treatments tended to decrease soil N_2_O emission rates when considered in isolation (−59%, −75% and −89%, respectively), but the combination of CO_2_, N, and burn treatments increased soil N_2_O emission rates by 958% (*P* = 0.04 for the Burn×CO_2_×N interaction).

### Microbial processes mediating the responses of soil N_2_O emissions to fire and global environmental changes

**Figure 3:**
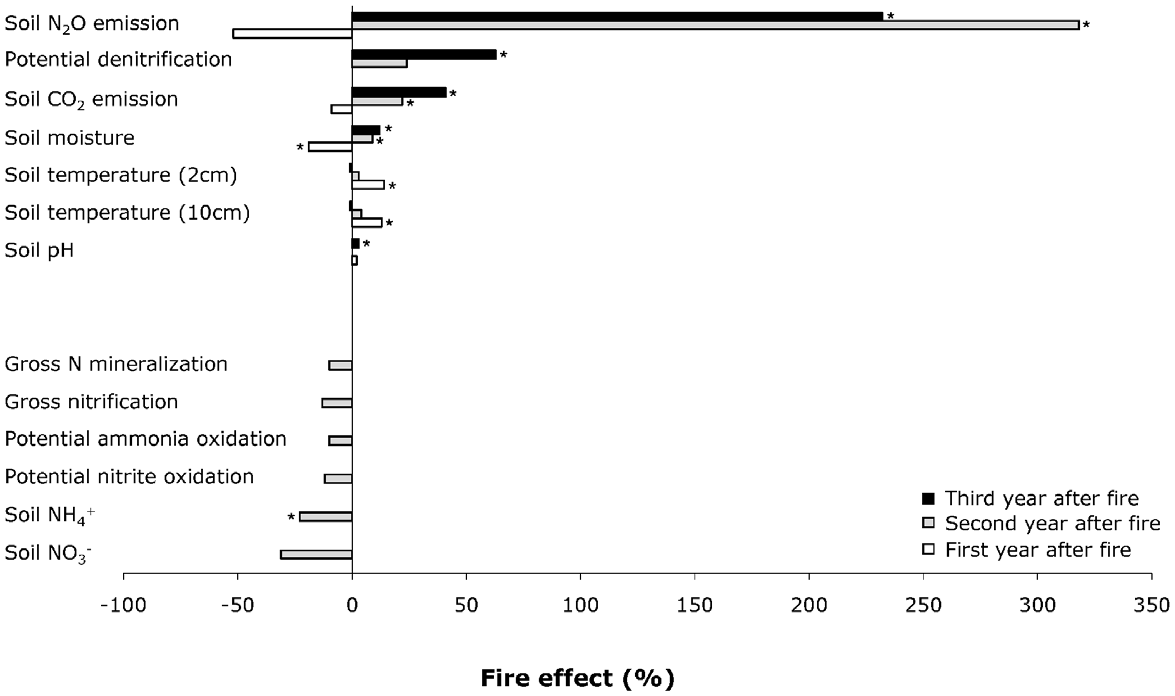
Effects of fire on soil N_2_O emissions, soil characteristics and soil nitrogen cycling. For each variable, bars are arranged chronologically from bottom to top: white bars correspond to data collected during first year after fire (i.e. 9 months after fire), grey bars to data collected for several dates during second year after fire (i.e. 15, 19 and 21 months after fire), and black bars to data collected during third year after fire (i.e. 33 months after fire). The top section shows the response to fire of soil N_2_O emissions, potential denitrification and soil characteristics measured over the three-year period following the fire (with the exception of year one for potential denitrification and of year two for soil pH). The bottom section shows the response to fire of soil N cycling variables measured for several dates in year two after fire. Fire effect sizes were calculated as: % effect = 100×[burned−unburned]/[unburned]. Asterisks next to bars indicate significant effects of fire (*P*<0.05).

To tease apart the microbial processes for the increases in soil N_2_O emission rates in the burned plots, especially when exposed to elevated levels of N and CO_2_, we investigated the responses of related N cycling processes to the treatments – including the responses of N mineralization, nitrification and denitrification – and the responses of the main drivers of these microbial processes. These drivers included soil ammonium and nitrate concentrations, soil environmental variables – soil moisture, soil temperature, soil pH - and soil CO_2_ emission rates, as an indicator of soil heterotrophic activity (Fig. 3, Tables S4, S5, S6, S7, S8, S9).

**Figure 4:**
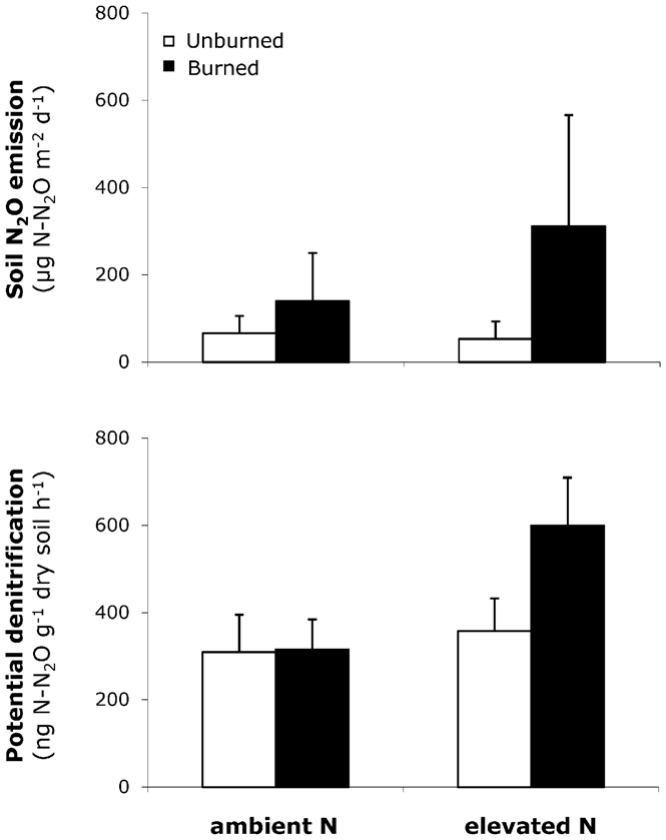
Effects of fire on soil N_2_O emission and potential denitrification rates under ambient and elevated nitrogen supply. The top section shows the effects of burn and N on soil N_2_O emission rates, and the bottom section shows the effects of burn and N on potential denitrification rates. The data of soil N_2_O emission and potential denitrification rates are grouped by burn treatment (unburned: open bars, burned: closed bars) and by N treatment (ambient vs. elevated). Each bar is the average of data collected 15, 19, 21, and 33 months after fire. Error bars denote pooled standard error (for each N treatment, n = 24×4 sampling dates for unburned plots; n = 16×4 sampling dates for burned plots).

Three lines of evidence indicated that increases in soil N_2_O emissions in the burned plots during the second and third years after fire were caused by higher denitrification rates. First, potential denitrification rates were higher in the burned compared to unburned plots (+37% on average, *P* = 0.03; Tables S4, S6 and S7; Fig. 3), whereas gross rates of N mineralization and gross and potential rates of nitrification either exhibited slightly negative responses or were non-responsive to fire (Tables S4, S8 and S9; Fig. 3). In particular, and in contrast to previous studies reporting increases in soil N_2_O emission rates after fire [22]–[23], [25], soil ammonium concentrations and nitrification rates were not increased in the burned compared to unburned plots (Tables S4, S8 and S9 and Fig. 3). Second, and consistent with this, soil N_2_O emission rates were significantly and positively correlated with potential denitrification rates (*P*<0.0001), and with well known drivers of denitrification [15]: soil moisture (*P*<0.0001) - an indicator of availability of anaerobic niches, and soil CO_2_ production rates (*P*<0.0001) - an indicator of labile C availability. Third, potential denitrification also exhibited a non-additive response to fire and enhanced N supply (*P*<0.0001 for the Burn×N interaction; Tables S6 and S7; Fig. 4). Thus, burn or elevated N alone had only small effects on potential denitrification (+2% and +15%, respectively), but the combination of burn and elevated N almost doubled potential denitrification (+94%). This interactive effect of fire and enhanced N supply on potential denitrification likely explained why increases in soil N_2_O emission rates were largest in the burned plots also exposed to elevated N (Fig. 4).

### Possible mechanisms underlying the responses of soil N_2_O emissions and denitrification to fire and global environmental changes

Our results suggest that the interactive responses of soil N_2_O emission and denitrification rates to fire and enhanced N supply (Fig. 4) resulted from the simultaneous relaxation of multiple constraints on denitrifying microorganisms in the burned and fertilized plots. In particular, denitrifying microorganisms may have benefited from decreased soil oxygen concentration and increased soil C and N availability in these plots.

Indeed, we found that the burn treatment significantly increased soil moisture during the second and third years after fire (by 9% year two after fire, *P* = 0.02, Table S6; by 12% year three after fire, *P* = 0.004, Table S7; Fig. 3). Resulting decreases in soil oxygen concentration likely stimulated anaerobic denitrification, as suggested by the significant positive relationship between soil moisture and potential denitrification rates (*P*<0.0001, R^2^ = 0.35), and as has been reported in other grassland studies [28], [30]. Increases in soil moisture in the burned plots may have resulted from post-fire decreases in evapotranspiration or increases in infiltration. Decreases in evapotranspiration in the burned plots during the second and third years after fire are however unlikely at our site. Indeed, fire increased plant production when combined with enhanced N supply and suppressed the negative effect of elevated CO_2_ on plant production at the end of the first year after the disturbance [8], but the responses of plant production to fire were short-lived and were no longer detected two or three years after fire [31]–[32]. Increases in soil moisture could thus reflect increases in infiltration in the burned plots resulting from changes in soil structure, e.g. from increases in soil aggregation and porosity as has been observed at some post-fire sites [33]. Post-fire increases in soil moisture may have enabled greater denitrification and N_2_O emission rates. However, other mechanisms must have been in play. Indeed, though elevated CO_2_ also significantly increased soil moisture (by 14% year three after fire, *P* = 0.009; Table S7), we observed no significant increases in denitrification or soil N_2_O emissions in the elevated CO_2_ plots (Tables S3, S6 and S7).

In addition to its effect on soil moisture, the burn treatment may have relaxed C limitation of denitrification [15]. This idea is in agreement with the increases in soil CO_2_ emission rates in the burned plots during the second and third years after fire (+22% year two after fire, *P* = 0.004, Table S6; +41% year three after fire, *P* = 0.009, Table S7; Fig. 3), suggesting higher soil labile C availability in these plots. This hypothesis is further supported by previous work at our site reporting decreases in the activity of extracellular enzymes involved in C acquisition in the burned plots during the second year after fire [9], though the response was not detected three years after fire [9]. Increased C availability in the burned plots, possibly reflecting increased C inputs to the soil from accelerated soil organic matter decomposition or stimulated primary productivity following burning [8], might have reduced microbial C demand and thereby activity of C acquisition enzymes [9]. Furthermore, the burn treatment may also have increased the availability of other nutrients (e.g. phosphorus (P)). Indeed, at the end of the first year after fire, decreased tissue N∶P ratio of grasses in the burned plots suggested increased P availability in these plots [8], [34].

Increases in soil N_2_O emission rates in response to fire were substantially larger when fire was combined with enhanced N supply and elevated CO_2_ (Fig. 2). In the burned plots exposed to elevated N, denitrifying microorganisms likely benefited from higher substrate N availability, as evidenced by large significant increases in soil nitrate concentrations (+771% on average, *P*<0.0001; Table S8) and in gross and potential nitrification rates (+72% on average, *P* = 0.003; Table S8 and +50% on average, *P*<0.0001, Table S9) in response to added N. Increases in denitrification and soil N_2_O emissions in the burned and fertilized plots (Fig. 4) thus probably resulted from increased soil moisture combined with greater C and N availability in these plots. The additional increase in soil N_2_O emission rates with elevated CO_2_ in the burned and fertilized plots (Fig. 2) could have resulted from further increases in soil moisture (Table S7) due to decreased plant transpiration [35]–[36] or from increases in soil temperature in the burned plots exposed to elevated CO_2_ (*P* = 0.04 for the Burn×CO_2_ interaction; Tables S6 and S7). Indeed, elevated CO_2_ reduced soil temperature in the unburned plots but increased soil temperature in the burned plots (+0.3°C at 2 cm depth; +0.5°C at 10 cm depth) which may have further stimulated denitrifying microorganisms, thus leading to even greater soil N_2_O emissions. In our experiment, it is unlikely that the increase in soil N_2_O emissions with elevated CO_2_ in the burned fertilized plots resulted solely from an increase in soil labile C. Indeed, and in contrast with other studies [37], elevated CO_2_ did not induce significant increases in root biomass production at our site [27], and even suppressed root allocation when combined with other treatments for some years [6]. Furthermore, elevated CO_2_ neither substantially increased soil CO_2_ emission rates (Tables S5, S6 and S7) nor substantially decreased extracellular enzyme activities involved in C acquisition [9].

### Responses of soil N_2_O emissions to fire over time

Soil N_2_O emissions responses to fire changed over time: soil N_2_O emissions were not significantly altered in the burned plots 9 months after fire, but were significantly increased in the burned plots during the second and third years after fire (Fig. 1).

The lack of increase in soil N_2_O emissions in the burned plots 9 months after fire is probably due to the decrease in soil moisture and absence of increased C availability in the burned plots at this time. Indeed, at the end of the first year after fire, fire significantly decreased soil moisture (by 19%, *P* = 0.01; Table S5; Fig. 3), presumably due to the removal of surface litter and to the subsequent increase in soil temperature [8], and did not significantly alter soil CO_2_ emission rates (Table S5) or extracellular enzyme activities [9]. Soil N_2_O emissions were then consistently higher in the burned compared to unburned plots and remained elevated by 232% in the burned plots almost 3 years after the fire (Fig. 1). Similarly, potential denitrification rates remained elevated 33 months after fire (+63%, *P* = 0.003; Table S7; Fig. 3), consistent with the microbial process invoked for the increase in N_2_O emissions in the burned plots. This long-lasting response of soil N_2_O emissions to the fire disturbance deserves attention. First, this response is more persistent than previously reported for chaparral or forest ecosystems, in which increased soil N_2_O emissions after fire lasted only several months and were associated with transient increases in soil inorganic N concentrations [22]–[25]. Second, this long-lasting response raises the question of whether increases in N_2_O production in the burned plots persisted beyond year three after fire. Soil moisture and soil CO_2_ emission rates remained significantly elevated in the burned plots 33 months after fire (+12%, *P* = 0.004 and +41%, *P* = 0.009; Table S7; Fig. 3). If the mechanisms proposed as responsible for increases in N_2_O production in the burned plots (i.e. greater soil moisture due to changes in soil properties and greater soil C or P availability) have persisted beyond year three after fire, or if other long-lasting mechanisms have occurred (e.g. post-fire shifts in the soil microbial communities, as observed by [38]), then increases in denitrification-associated N_2_O efflux may have lasted even longer. This is especially true for the burned fertilized plots, where N inputs could have sustained increased soil N_2_O emissions.

### Conclusions and implications

Our study provides evidence that global environmental changes could strongly amplify fire effects on soil N_2_O production. We found that over a three-year period following a fire in a California grassland, soil N_2_O emission rates were increased by a factor of two in the burned plots (+124%) and by a factor of six in the burned plots exposed to elevated CO_2_ and enhanced N supply (+516%). We also provide evidence that the underlying mechanism for this response was increased microbial denitrification, resulting from increased soil moisture and soil C and N availability in the burned and fertilized plots. The responses of soil N_2_O emission rates to fire were large and persisted for at least three years after the fire. Thus, our results clearly stress the need to further explore the interactions between fire disturbances and global environmental changes. First, they indicate that limiting global change studies to undisturbed ecosystems could underestimate the impacts global environmental changes may have on soil greenhouse gas production. Second, they suggest that the interactive effects of fire and global environmental changes could play a significant role in controlling the greenhouse gas balance of grassland ecosystems and promote them as significant sources of N_2_O.

## Materials and Methods

### The study site and experimental design

This study took place at the Jasper Ridge Global Change Experiment (JRGCE) in the Jasper Ridge Biological Preserve, which is located in the eastern foothills of the Santa Cruz Mountains in northern California (37°24′N, 122°14′W). The site experiences a Mediterranean-type climate with a cool, wet growing season from November to March, and a hot, dry summer from June to October. The dominant species are annual grasses (especially *Avena barbata* and *Bromus hordeaceus*) and annual forbs (especially *Geranium dissectum* and *Erodium botrys*) [26]. The soil is a fine, mixed, Typic Haploxeralf developed from Franciscan complex alluvium sandstone [9].

The JRGCE was initiated in November 1998 and provides a complete factorial design of four treatments at two levels (ambient vs. elevated): atmospheric CO_2_ concentration (ambient vs. 680 µmol mol^−1^), temperature (ambient vs. soil surface warming of 0.8–1.0°C), precipitation (ambient vs. +50% above ambient), and N supply (ambient vs. +7 g N m^−2^ yr^−1^) [6], [26]–[27]. These treatments were selected to mimic conditions predicted to occur at the end of the 21^st^ century for central California, and to allow a more comprehensive understanding of mechanisms driving the grassland responses to future changes in the environment [6], [26]–[27]. CO_2_ is elevated with a free air CO_2_ enrichment (FACE) system delivering pure CO_2_ at the plant height. Temperature is increased using overhead infrared heaters. Precipitation was enhanced at first with drip irrigation (1998–2000) and then with overhead sprinklers (2001–2006). N is applied twice per year as Ca(NO_3_)_2_, with an initial application of 2 g N m^−2^ in solution early in the growing season (each November) and an additional application of 5 g N m^−2^ as slow-release fertilizer (Nutricote 12-0-0, Agrivert, Riverside, CA, USA) later in the growing season (each January) [27]. Treatments are organized in a randomized block split-plot design, with CO_2_ and temperature treatments applied at the plot level (in 2 m-diameter circular plots) and precipitation and N treatments applied at the subplot level (in 0.78 m^2^-quadrants – each plot being divided in four quadrants with 0.5 m solid belowground barriers and mesh aboveground partitions).

Each of the 16 possible treatment combinations was initially replicated eight times. However, in July 2003, an accidental, rapid and low-intensity fire ashed all aboveground litter in two of the eight blocks of the experiment [8]–[9]. Warming was discontinued in the burned plots immediately after the fire, thus leading to a new complete factorial design with four “treatments” at two levels: burn (unburned vs. burned), CO_2_, precipitation and N supply (ambient vs. elevated). Combinations of the CO_2_, precipitation and N treatments were replicated six times in the unburned plots and four times in the burned plots [8]–[9].

### Soil N_2_O and CO_2_ emission rates

Soil N_2_O and CO_2_ emission rates were measured during the three years following the fire disturbance: at the end of each of the three growing seasons (i.e. at the time of peak biomass of plants: 9, 21 and 33 months after fire), and for multiple dates during the second growing season after fire (at early germination: 15 months after fire; at mid vegetative stage: 19 months after fire). Measurements performed 15 months after fire were conducted at four dates following a simulated 20-mm rainfall event marking the end of the dry season (an average of data collected was used for analysis); measurements performed 19 months after fire were conducted at two dates (an average of data collected was used for analysis).

Measurements were made using a static chamber approach [39], with chambers (1.8 L/V) constructed from 10.2 cm-diameter PVC pipe closed with a PVC cap. At each measurement date, chambers were placed in each quadrant into a permanent respiration ring. Three subsequent 15 mL-headspace air samples were then taken at 15-minute intervals through a septum installed at the top of each chamber using a nylon syringe. Samples were then analyzed for N_2_O and CO_2_ concentrations on a gas chromatograph system (Agilent 6890 GC System, Palo Alto, CA) with Haysep Q 60/80 and Porapack Q 60/80 packed columns, equipped with an electron capture detector to determine N_2_O concentrations and a flame-ionization detector with a methanizer to determine CO_2_ concentrations. Field N_2_O and CO_2_ emission rates were calculated using linear regression analysis of concentrations over time.

### Soil sampling

Soil cores (5 cm diameter×5 cm deep) were sampled in each quadrant at each date where soil N_2_O emission rates were measured, i.e. 9, 15, 19, 21, and 33 months after fire. Soil samples collected 15 months after fire were collected prior to the simulated rainfall event. In addition, soil cores (2.2 cm diameter×15 cm deep) were collected 3 months before fire (i.e. at the end of the last growing season preceding the wildfire) and were used as control samples of the pre-burn conditions [28]. At each sampling date, large roots and rocks were removed by hand, and soil sample heterogeneity was reduced by thorough mixing. Soil samples were used for measurements of soil environmental variables (soil moisture, soil pH and soil NH_4_^+^ and NO_3_^−^concentrations), as well as for measurements of gross or potential rates of N mineralization, nitrification and denitrification.

### Soil environmental variables

Gravimetric soil moisture was determined at each soil sampling date by comparing the mass of a 5-g soil sample before and after drying at 105°C. Soil temperature data were obtained at hourly intervals from thermocouples buried at 2 cm and 10 cm below the soil surface in each quadrant, and averaged over each soil N_2_O efflux measurement date. Soil pH was measured in 1∶1 mixture of soil and distilled water on soil samples collected during year one and year three after fire (i.e. 9 and 33 months after fire). Soil NH_4_^+^ and NO_3_^−^ concentrations were measured on soil samples collected during year two after fire (i.e. 15, 19 and 21 months after fire). At each date, NH_4_^+^ and NO_3_^−^ were extracted in 25 mL of 0.25 M K_2_SO_4_ from 10 g soil samples, which were vigorously shaken for 30 min. Extracts were filtered and analyzed colorimetrically for NH_4_^+^ and NO_3_^−^ concentrations using an autoanalyzer (Lachat Quickchem FIA+8000).

### Gross rates of N mineralization and nitrification

Gross N mineralization and nitrification rates were determined using ^15^N pool dilution [40] on soil samples collected during year two after fire (i.e. 19 and 21 months after fire for gross N mineralization; 15, 19 and 21 months after fire for gross nitrification). At each date, two 50-g soil samples from each quadrant were placed in separate plastic bags, and 3 mL of either ^15^N-(NH_4_)_2_SO_4_ or ^15^N-Ca(NO_3_)_2_ were added (99 atom % ^15^N) and thoroughly mixed to each, producing target concentrations of 1 µg ^15^N g^−1^ dry soil. One 10-g soil subsample was immediately taken and extracted with 25 mL 0.25 M K_2_SO_4_ for determination of the initial NH_4_^+^ and NO_3_^−^ concentrations. After a 24 h incubation period in the field, a second 10-g subsample was taken and equally extracted. Extracts were filtered and analyzed colorimetrically for NH_4_^+^ and NO_3_^−^ concentrations using an autoanalyzer (Lachat Quickchem FIA+8000). A diffusion procedure onto acidified filter disks was used to separate NH_4_^+^ and NO_3_^−^ in soil extracts [41] and filter disks were then analyzed for ^15^N-NH_4_^+^ and ^15^N-NO_3_^−^ contents by isotope ratio mass spectrometry at the Colorado Plateau Stable Isotope Laboratory (http://www.mpcer.nau.edu/isotopelab/). Gross N mineralization rates were calculated based on NH_4_^+^ and ^15^N-NH_4_^+^ concentrations at time 0 and time 24, and gross nitrification rates based on NO_3_^−^ and ^15^N-NO_3_^−^ concentrations at time 0 and time 24, according to ^15^N pool dilution equations [40].

### Potential rates of nitrification and denitrification

Potential nitrification and denitrification rates were measured on soil samples collected 3 months prior to the fire (data prior to the fire are from [28]) and on soil samples collected during year two and year three after fire (i.e. 19 and 21 months after fire for potential nitrification; 15, 19, 21 and 33 months after fire for potential denitrification). Measurements of potential rates of nitrification and denitrification are proxies of measurements of the concentrations of the nitrifying and denitrifying enzymes in soils [40], [42]. These concentrations of enzymes (i) are determined by the *in situ* environmental constraints to which nitrifying and denitrifying microorganisms were exposed in the field prior to soil sampling; and (ii) are measured in laboratory incubations during which substrates are made non-limiting and environmental conditions are made optimal for the reaction considered, over time periods where *de novo* synthesis of enzymes does not occur [42]–[44]. Measurements of potential rates of nitrification and denitrification thus reflect the direction and magnitude of the environmental constraints in the field on the nitrifying and denitrifying microorganisms. Potential rates of nitrification and denitrification are thought to be more constant over time than *in situ* rates of nitrification and denitrification [44]–[45], and have been widely used to provide information on the impacts of environmental changes on the size of the nitrifying and denitrifying microbial communities.

Potential rates of nitrification were measured on soil samples collected prior to the fire according to [28]. Both potential rates of ammonia oxidation and nitrite oxidation – the two distinct steps of nitrification – were measured on soil samples collected year two after fire. Potential ammonia oxidation rates were measured as NO_2_^−^ production rates from soil samples amended with NH_4_^+^ and NaClO_3_, an inhibitor of the microbial oxidation of NO_2_^−^ into NO_3_^−^ [46]: 5 g equivalent dry soil were supplied with 50 mL of a solution of 0.18 mM (NH_4_)_2_SO_4_, 0.8 mM K_2_HPO_4_, 0.1 mM KH_2_PO_4_ and 0.01 M NaClO_3_, and were incubated at 28°C for 9 h with agitation at 150 rpm. NO_2_^−^ concentration was measured after 0 h, 3 h, 6 h and 9 h on a spectrophotometer (Uvikon 800, Leeds, UK) at 520 nm using the Griess reagent. Rates of NO_2_^−^ production were constant during ammonia oxidation assays (data not shown). Potential nitrite oxidation rates were measured as NO_2_^−^ consumption rates from soil samples amended with NO_2_^−^ [47]: 5 g equivalent dry soil were supplied with 50 mL of a solution of 0.36 mM NaNO_2_ and were incubated at 28°C for 30 h with agitation at 150 rpm. NO_2_^−^ concentration was measured after 0 h, 9 h, 24 h and 30 h as described above. Rates of NO_2_^−^ consumption were constant during nitrite oxidation assays (data not shown).

Potential denitrification rates were measured on soil samples collected prior to the fire [28] and on soil samples collected year two and year three after fire as N_2_O production rates from soil samples amended with NO_3_^−^ and labile C, and in which N_2_O reductase was inhibited with acetylene [48]: 5 g equivalent dry soil were placed in 150 mL plasma flasks sealed with rubber stoppers and amended with 1 mg C-glucose g^−1^ dry soil, 1 mg C-glutamic acid g^−1^ dry soil and 0.1 mg N-NO_3_^−^ g^−1^ dry soil. The atmosphere of the flask was replaced by a He∶C_2_H_2_ mixture (90∶10) to ensure anaerobic conditions and inhibition of N_2_O reductase. Flasks were incubated at 27°C for 8 h. N_2_O concentration was measured every two hours on a gas chromatograph equipped with an electron capture detector (Agilent Micro GC, P200). Rates of N_2_O production were constant during denitrification assays (data not shown).

### Plant and litter biomass

End-season plant and litter biomass were measured at the end of the growing season preceding the fire (i.e. 3 months before fire) and at the end of the three growing seasons following the fire (i.e. 9, 21 and 33 months after fire). At each measurement date, all aboveground plant matter was collected in a 141 cm^2^ area and separated into live and litter material, and root biomass was determined by separating live roots out of 15 cm-depth soil cores taken in the area of the aboveground biomass harvest [8], [27]. Aboveground biomass, senesced aboveground tissue and belowground biomass were oven-dried at 70°C before weighing. Total plant biomass was estimated as the sum of above- and belowground biomass, and litter biomass as the biomass of senesced aboveground tissue. Data on biomass prior to the fire are from [27]; data 9 months after the fire are from [8]; preliminary data 21 and 33 months after the fire are available on request from Nona Chiariello (nonajrbp@stanford.edu).

### Statistical analysis

All statistical analyses were performed using SAS 9.2 (SAS Institute, Cary, NC, USA). We analyzed our data with PROC MIXED using a repeated four-way split-plot analysis of variance in order to assess the overall effects of the burn and other global environmental changes treatments on soil N_2_O emissions over the three year-period following the fire, as well as the temporal variability of these treatment effects. The burn and CO_2_ treatments were included as whole-plot effects, while the precipitation and N treatments were included as split-plot effects. We also analyzed our data for each individual measurement date using a full factorial split-plot mixed model in order to assess the treatment effects at each date. As the responses of soil N_2_O emissions to the burn treatment differed depending on year since fire, we analyzed the effects of the treatments on the other variables for each individual year after fire. Data collected year one or year three after fire were analyzed using a full factorial split-plot mixed model. Data collected year two after fire were analyzed using a repeated four-way split-plot analysis of variance (all measurement dates were included, except for soil moisture for which data collected 15 months after fire were excluded from the analysis as measurements of soil N_2_O emissions were performed following a wet-up experiment, while measurements of soil moisture were conducted on soil samples collected prior to the wet-up experiment). In addition, we analyzed the data collected 3 months before fire using the same full factorial split-plot model to verify that prior to the fire, microbial and plant characteristics were indistinguishable between the plots that later burned and those that did not (Table S1). We also analyzed the response of the soil N_2_O emissions to the “previously warmed” treatment in the burned plots by carrying out analyses of variance with PROC MIXED to verify that soil N_2_O emissions were indistinguishable between the previously warmed burned plots and the previously un-warmed burned plots (Table S2). Data were square-root or log transformed prior to analysis to correct non-homogeneity of variance (a square-root transformation was used for soil N_2_O and CO_2_ emission rates and potential nitrification; a log transformation was used for other variables). Effects with *P*<0.05 are referred to as significant.

Finally, we performed correlation analyses between soil N_2_O emission rates, rates of microbial processes contributing to N_2_O production in soils (i.e. gross or potential rates of nitrification and denitrification), and main drivers of these microbial processes (i.e. soil environmental variables, gross N mineralization and soil CO_2_ emission rates) to provide insights into the mechanisms controlling the response of the soil N_2_O emissions to the treatments. Given the large number of correlations performed, we applied Bonferroni corrections by dividing α (α = 0.05) by the number n of correlations (n = 11) and by checking significance at *P*<α/n (i.e. *P*<0.0045).

## Supporting Information

Table S1 Treatment effects on potential nitrification and denitrification, and litter and plant biomass prior to the fire (n = 80). Treatments are elevated CO_2_ (CO_2_), increased precipitation (W) and N supply (N). The treatment “B” (plots that burned in July 2003) was included in the analysis to verify that the variables measured in April 2003 were indistinguishable between the plots that later burned and those that did not. Significant responses are indicated in bold (α = 0.05). As shown in the table, prior to the fire, no measured variable was significantly different between the plots that later burned and those that remained untouched by the fire (“B”: *P*>0.05 in all cases). Data of potential N rates prior to the fire are from [28]; data of plant biomass prior to the fire are from [27].
(DOC)

Table S2 Effect of the “previously warmed” treatment on soil N_2_O emission rates in the burned plots (n = 32 for each measurement date). As shown in the table, for each measurement date, soil N_2_O emission rates were not significantly different between the previously warmed burned plots and the previously un-warmed burned plots (*P*>0.05 in all cases).
(DOC)

Table S3 Treatment effects on soil N_2_O emission rates over the three years following the fire (n = 80×5 sampling dates – 9, 15, 19, 21 and 33 months after fire). Treatments are burn (B), elevated CO_2_ (CO_2_), increased precipitation (W), and N supply (N). Significant responses are indicated in bold (α = 0.05). The overall effect of the burn treatment was calculated as: % effect = 100×[burned−unburned]/unburned (n = 32×5 in the burned plots, n = 48×5 in the unburned plots). The overall effects of the CO_2_, precipitation, and N treatments were calculated as: % effect = 100×[elevated−ambient]/ambient (n = 40×5 in the elevated and ambient plots).
(DOC)

Table S4 Mean values of soil N_2_O and CO_2_ emission rates and soil N cycling variables in the unburned and burned plots. For each variable, data were grouped by burn treatment (unburned vs. burned) and averaged across the CO_2_, precipitation and nitrogen treatments. Values indicate mean (averaged across all available measurement dates) ± pooled standard error. The number of data in the unburned and burned plots multiplied by the number of measurement dates for each variable is indicated in parentheses. Refer to Tables S3 and Tables S5 to S9 for the results from mixed model analysis testing for a fire effect on the variables presented in Table S4.
(DOC)

Table S5 Treatment effects on soil CO_2_ emission rates, soil moisture and soil temperature (at 2 cm depth) year one after fire (n = 80). Treatments are burn (B), elevated CO_2_ (CO_2_), increased precipitation (W), and N supply (N). Significant responses are indicated in bold (α = 0.05). The overall effect of the burn treatment was calculated as: % effect = 100×[burned−unburned]/unburned (n = 32 in the burned plots, n = 48 in the unburned plots). The overall effects of the CO_2_, precipitation, and N treatments were calculated as: % effect = 100×[elevated−ambient]/ambient (n = 40 in the elevated and ambient plots).
(DOC)

Table S6 Treatment effects on potential denitrification, soil CO_2_ emission rates, soil moisture and soil temperature (at 2 cm depth) year two after fire (n = 80×3 sampling dates – 15, 19 and 21 months after fire, except for soil moisture where n = 80×2 sampling dates – 19 and 21 months after fire). Treatments are burn (B), elevated CO_2_ (CO_2_), increased precipitation (W), and N supply (N). Significant responses are indicated in bold (α = 0.05). The overall effect of the burn treatment was calculated as: % effect = 100×[burned−unburned]/unburned (n = 32×3 in the burned plots, n = 48×3 in the unburned plots). The overall effects of the CO_2_, precipitation, and N treatments were calculated as: % effect = 100×[elevated−ambient]/ambient (n = 40×3 in the elevated and ambient plots).
(DOC)

Table S7 Treatment effects on potential denitrification, soil CO_2_ emission rates, soil moisture and soil temperature (at 2 cm depth) year three after fire (n = 80). Treatments are burn (B), elevated CO_2_ (CO_2_), increased precipitation (W), and N supply (N). Significant responses are indicated in bold (α = 0.05). The overall effect of the burn treatment was calculated as: % effect = 100×[burned−unburned]/unburned (n = 32 in the burned plots, n = 48 in the unburned plots). The overall effects of the CO_2_, precipitation, and N treatments were calculated as: % effect = 100×[elevated−ambient]/ambient (n = 40 in the elevated and ambient plots).
(DOC)

Table S8 Treatment effects on gross N mineralization, gross nitrification and soil NH_4_^+^ and NO_3_^−^ concentrations year two after fire (n = 80×3 sampling dates – 15, 19 and 21 months after fire, except for N mineralization where n = 80×2 sampling dates – 19 and 21 months after fire). Treatments are burn (B), elevated CO_2_ (CO_2_), increased precipitation (W), and N supply (N). Significant responses are indicated in bold (α = 0.05). The overall effect of the burn treatment was calculated as: % effect = 100×[burned−unburned]/unburned (n = 32×3 in the burned plots, n = 48×3 in the unburned plots). The overall effects of the CO_2_, precipitation, and N treatments were calculated as: % effect = 100×[elevated−ambient]/ambient (n = 40×3 in the elevated and ambient plots).
(DOC)

Table S9 Treatment effects on potential ammonia and nitrite oxidation year two after fire (n = 80×2 sampling dates – 19 and 21 months after fire). Treatments are burn (B), elevated CO_2_ (CO_2_), increased precipitation (W), and N supply (N). Significant responses are indicated in bold (α = 0.05). The overall effect of the burn treatment was calculated as: % effect = 100×[burned−unburned]/unburned (n = 32×2 in the burned plots, n = 48×2 in the unburned plots). The overall effects of the CO_2_, precipitation, and N treatments were calculated as: % effect = 100×[elevated−ambient]/ambient (n = 40×2 in the elevated and ambient plots).
(DOC)
