# Supplementary material for: Global Change Could Amplify Fire Effects on Soil Greenhouse Gas Emissions
Source: PLoS One. 2011 Jun 8;6(6):e20105. doi: 10.1371/journal.pone.0020105 (PMC3110610; doi:10.1371/journal.pone.0020105)
Supplement: Table S1 — Treatment effects on potential nitrification and denitrification, and litter and plant biomass prior to the fire (n = 80). Treatments are elevated CO2 (CO2), increased precipitation (W) and N supply (N). The treatment “B” (plots that burned in July 2003) was included in the analysis to verify that the variables measured in April 2003 were indistinguishable between the plots that later burned and those that did not. Significant responses are indicated in bold (α = 0.05). As shown in the table, prior to the fire, no measured variable was significantly different between the plots that later burned and those that remained untouched by the fire (“B”: P>0.05 in all cases). Data of potential N rates prior to the fire are from [28]; data of plant biomass prior to the fire are from [27]. (DOC) [file pone.0020105.s001.doc]

**Table S1. Treatment effects on potential nitrification and denitrification, and litter and plant biomass prior to the fire (n = 80)**

|  | **Potential nitrification** | **Potential denitrification** | **Litter** | **Plant biomass** |
| --- | --- | --- | --- | --- |
| **Treatment** | p-value | p-value | p-value | p-value |
| **“B”** | 0.18 | 0.23 | 0.17 | 0.14 |
| **CO_2_** | **0.004** | 0.38 | 0.70 | 0.33 |
| **W** | 0.68 | **0.01** | 0.39 | 0.08 |
| **N** | **0.05** | **0.001** | 0.11 | **0.03** |
| **“B” x CO_2_** | 0.93 | 0.47 | 0.12 | 0.60 |
| **“B” x W** | 0.53 | 0.47 | 0.31 | 0.42 |
| **“B” x N** | 0.38 | 0.71 | 0.26 | 0.53 |
| **CO_2_ x W** | 0.96 | 0.87 | 0.10 | 0.38 |
| **CO_2_ x N** | **0.002** | **0.002** | 0.94 | 0.61 |
| **W x N** | 0.28 | 0.37 | 0.98 | 0.80 |
| **“B” x CO_2_ x W** | 0.52 | 0.51 | 0.80 | 0.07 |
| **“B” x CO_2_ x N** | 0.71 | 0.24 | 0.84 | 0.29 |
| **“B” x W x N** | 0.74 | 0.67 | 0.78 | 0.39 |
| **CO_2_ x W x N** | 0.28 | 0.97 | 0.63 | 0.85 |
| **“B” x CO_2_ x W x N** | 0.72 | 0.92 | 0.50 | 0.39 |

Treatments are elevated CO_2_ (CO_2_), increased precipitation (W) and N supply (N). The treatment “B” (plots that burned in July 2003) was included in the analysis to verify that the variables measured in April 2003 were indistinguishable between the plots that later burned and those that did not. Significant responses are indicated in bold (α = 0.05). As shown in the table, prior to the fire, no measured variable was significantly different between the plots that later burned and those that remained untouched by the fire (“B”: *P* > 0.05 in all cases). Data of potential N rates prior to the fire are from [28]; data of plant biomass prior to the fire are from [27].
