# Supplementary material for: Global Change Could Amplify Fire Effects on Soil Greenhouse Gas Emissions
Source: PLoS One. 2011 Jun 8;6(6):e20105. doi: 10.1371/journal.pone.0020105 (PMC3110610; doi:10.1371/journal.pone.0020105)
Supplement: Table S2 — Effect of the “previously warmed” treatment on soil N2O emission rates in the burned plots (n = 32 for each measurement date). As shown in the table, for each measurement date, soil N2O emission rates were not significantly different between the previously warmed burned plots and the previously un-warmed burned plots (P>0.05 in all cases). (DOC) [file pone.0020105.s002.doc]

**Table S2. Effect of the “previously warmed” treatment on soil N_2_O emission rates in the burned plots (n = 32 for each measurement date)**

|  | **Time since fire** | | | | |
| --- | --- | --- | --- | --- | --- |
|  | **9 months** | **15 months** | **19 months** | **21 months** | **33 months** |
| **Treatment** | p-value | p-value | p-value | p-value | p-value |
| **“Previously warmed”** | 0.74 | 0.95 | 0.25 | 0.12 | 0.59 |

As shown in the table, for each measurement date, soil N_2_O emission rates were not significantly different between the previously warmed burned plots and the previously un-warmed burned plots (*P* > 0.05 in all cases).
