# Supplementary material for: Global Change Could Amplify Fire Effects on Soil Greenhouse Gas Emissions
Source: PLoS One. 2011 Jun 8;6(6):e20105. doi: 10.1371/journal.pone.0020105 (PMC3110610; doi:10.1371/journal.pone.0020105)
Supplement: Table S3 — Treatment effects on soil N2O emission rates over the three years following the fire (n = 80×5 sampling dates – 9, 15, 19, 21 and 33 months after fire). Treatments are burn (B), elevated CO2 (CO2), increased precipitation (W), and N supply (N). Significant responses are indicated in bold (α = 0.05). The overall effect of the burn treatment was calculated as: % effect = 100×[burned−unburned]/unburned (n = 32×5 in the burned plots, n = 48×5 in the unburned plots). The overall effects of the CO2, precipitation, and N treatments were calculated as: % effect = 100×[elevated−ambient]/ambient (n = 40×5 in the elevated and ambient plots). (DOC) [file pone.0020105.s003.doc]

**Table S3. Treatment effects on soil N_2_O emission rates over the three years following the fire (n = 80 x 5 sampling dates – 9, 15, 19, 21 and 33 months after fire)**

|  | **Soil N_2_O emission** | |
| --- | --- | --- |
| **Treatment** | % effect | p-value |
| **B** | **227** | **0.001** |
| **CO_2_** | 35 | 0.40 |
| **W** | -9 | 0.83 |
| **N** | **63** | **0.02** |
| **B x CO_2_** |  | 0.65 |
| **B x W** |  | 0.52 |
| **B x N** |  | **0.01** |
| **CO_2_ x W** |  | 0.83 |
| **CO_2_ x N** |  | 0.17 |
| **W x N** |  | 0.97 |
| **B x CO_2_ x W** |  | 0.52 |
| **B x CO_2_ x N** |  | 0.36 |
| **B x W x N** |  | 0.85 |
| **CO_2_ x W x N** |  | 0.20 |
| **B x CO_2_ x W x N** |  | 0.28 |
|  |  |  |
| **Time** |  | **<0.0001** |
| **Time x B** |  | **0.004** |
| **Time x CO_2_** |  | 0.05 |
| **Time x W** |  | 0.05 |
| **Time x N** |  | **0.01** |
| **Time x B x CO_2_** |  | **0.0007** |
| **Time x B x W** |  | 0.89 |
| **Time x B x N** |  | **0.0001** |
| **Time x CO_2_ x W** |  | 0.10 |
| **Time x CO_2_ x N** |  | **0.009** |
| **Time x W x N** |  | 0.79 |
| **Time x B x CO_2_ x W** |  | 0.23 |
| **Time x B x CO_2_ x N** |  | **0.0007** |
| **Time x B x W x N** |  | 0.50 |
| **Time x CO_2_ x W x N** |  | 0.78 |
| **Time x B x CO_2_ x W x N** |  | 0.90 |

Treatments are burn (B), elevated CO_2_ (CO_2_), increased precipitation (W), and N supply (N). Significant responses are indicated in bold (α = 0.05). The overall effect of the burn treatment was calculated as: % effect = 100 x [burned – unburned] / unburned (n = 32 x 5 in the burned plots, n = 48 x 5 in the unburned plots). The overall effects of the CO_2_, precipitation, and N treatments were calculated as: % effect = 100 x [elevated – ambient] / ambient (n = 40 x 5 in the elevated and ambient plots).
