# Supplementary material for: Global Change Could Amplify Fire Effects on Soil Greenhouse Gas Emissions
Source: PLoS One. 2011 Jun 8;6(6):e20105. doi: 10.1371/journal.pone.0020105 (PMC3110610; doi:10.1371/journal.pone.0020105)
Supplement: Table S4 — Mean values of soil N2O and CO2 emission rates and soil N cycling variables in the unburned and burned plots. For each variable, data were grouped by burn treatment (unburned vs. burned) and averaged across the CO2, precipitation and nitrogen treatments. Values indicate mean (averaged across all available measurement dates) ± pooled standard error. The number of data in the unburned and burned plots multiplied by the number of measurement dates for each variable is indicated in parentheses. Refer to Tables S3 and Tables S5 to S9 for the results from mixed model analysis testing for a fire effect on the variables presented in Table S4. (DOC) [file pone.0020105.s004.doc]

**Table S4. Mean values of soil N_2_O and CO_2_ emission rates and soil N cycling variables in the unburned and burned plots**

|  | **Unburned** | **Burned** |
| --- | --- | --- |
| **Variable** | **Mean ± pooled se (n)** | **Mean ± pooled se (n)** |
|  | | |
| **Soil N_2_O emission**  µg N-N_2_O m^-2^ d^-1^ | 56 ± 30 (48 x 5) | 185 ± 145 (32 x 5) |
|  | | |
| **Soil CO_2_ emission**  g C-CO_2_ m^-2^ d^-1^ | 3.5 ± 0.6 (48 x 5) | 4.3 ± 0.8 (32 x 5) |
|  |  |  |
| **Potential denitrification**  ng N-N_2_O g^-1^ dry soil h^-1^ | 333 ± 58 (48 x 4) | 458 ± 88 (32 x 4) |
|  |  |  |
| **Gross N mineralization**  ng N-NH_4_^+^ g^-1^ dry soil h^-1^ | 170 ± 17 (48 x 2) | 153 ± 17 (32 x 2) |
|  |  |  |
| **Gross nitrification**  ng N-NO_3_^-^ g^-1^ dry soil h^-1^ | 205 ± 61 (48 x 3) | 178 ± 43 (32 x 3) |
|  |  |  |
| **Soil NH_4_^+^ concentrations**  µg N-NH_4_^+^ g^-1^ dry soil | 6.3 ± 1.2 (48 x 3) | 4.9 ± 1.1 (32 x 3) |
|  |  |  |
| **Soil NO_3_^-^ concentrations**  µg N-NO_3_^-^ g^-1^ dry soil | 9.9 ± 5.3 (48 x 3) | 6.8 ± 4.7 (32 x 3) |
|  |  |  |
| **Potential ammonia oxidation**  ng N-NO_2_^-^ g^-1^ dry soil h^-1^ | 86 ± 9 (48 x 2) | 78 ± 11 (32 x 2) |
|  | | |
| **Potential nitrite oxidation**  ng N-NO_3_^-^ g^-1^ dry soil h^-1^ | 527 ± 47 (48 x 2) | 463 ± 46 (32 x 2) |
|  | | |

For each variable, data were grouped by burn treatment (unburned vs. burned) and averaged across the CO_2_, precipitation and nitrogen treatments. Values indicate mean (averaged across all available measurement dates) ± pooled standard error. The number of data in the unburned and burned plots multiplied by the number of measurement dates for each variable is indicated in parentheses. Refer to Tables S3 and Tables S5 to S9 for the results from mixed model analysis testing for a fire effect on the variables presented in Table S4.
