# Supplementary material for: Global Change Could Amplify Fire Effects on Soil Greenhouse Gas Emissions
Source: PLoS One. 2011 Jun 8;6(6):e20105. doi: 10.1371/journal.pone.0020105 (PMC3110610; doi:10.1371/journal.pone.0020105)
Supplement: Table S5 — Treatment effects on soil CO2 emission rates, soil moisture and soil temperature (at 2 cm depth) year one after fire (n = 80). Treatments are burn (B), elevated CO2 (CO2), increased precipitation (W), and N supply (N). Significant responses are indicated in bold (α = 0.05). The overall effect of the burn treatment was calculated as: % effect = 100×[burned−unburned]/unburned (n = 32 in the burned plots, n = 48 in the unburned plots). The overall effects of the CO2, precipitation, and N treatments were calculated as: % effect = 100×[elevated−ambient]/ambient (n = 40 in the elevated and ambient plots). (DOC) [file pone.0020105.s005.doc]

**Table S5** Treatment effects on soil CO_2_ emission rates, soil moisture and soil temperature (at 2 cm depth) year one after fire (n = 80)

|  | **Soil CO_2_ emission** | | **Soil moisture** | | **Soil temperature** | |
| --- | --- | --- | --- | --- | --- | --- |
| **Treatment** | % effect | p-value | % effect | p-value | % effect | p-value |
| **B** | -9 | 0.64 | **-19** | **0.01** | **14** | **0.0001** |
| **CO_2_** | 23 | 0.27 | **22** | **0.03** | -1 | 0.99 |
| **W** | 20 | 0.25 | **23** | **0.0001** | -1 | 0.61 |
| **N** | 1 | 0.67 | -7 | 0.12 | **-3** | **0.02** |
| **B x CO_2_** |  | 0.17 |  | 0.14 |  | 0.13 |
| **B x W** |  | 0.28 |  | 0.64 |  | 0.71 |
| **B x N** |  | 0.90 |  | 0.17 |  | 0.34 |
| **CO_2_ x W** |  | 0.87 |  | 0.29 |  | 0.80 |
| **CO_2_ x N** |  | 0.17 |  | 0.81 |  | 0.68 |
| **W x N** |  | 0.64 |  | 0.56 |  | 0.85 |
| **B x CO_2_ x W** |  | 0.49 |  | 0.71 |  | 0.32 |
| **B x CO_2_ x N** |  | 0.25 |  | 0.67 |  | 0.29 |
| **B x W x N** |  | 0.12 |  | 0.47 |  | 0.86 |
| **CO_2_ x W x N** |  | 0.48 |  | 0.22 |  | 0.66 |
| **B x CO_2_ x W x N** |  | 0.88 |  | 0.94 |  | 0.54 |

Treatments are burn (B), elevated CO_2_ (CO_2_), increased precipitation (W), and N supply (N). Significant responses are indicated in bold (α = 0.05). The overall effect of the burn treatment was calculated as: % effect = 100 x [burned – unburned] / unburned (n = 32 in the burned plots, n = 48 in the unburned plots). The overall effects of the CO_2_, precipitation, and N treatments were calculated as: % effect = 100 x [elevated – ambient] / ambient (n = 40 in the elevated and ambient plots).
