# Supplementary material for: Global Change Could Amplify Fire Effects on Soil Greenhouse Gas Emissions
Source: PLoS One. 2011 Jun 8;6(6):e20105. doi: 10.1371/journal.pone.0020105 (PMC3110610; doi:10.1371/journal.pone.0020105)
Supplement: Table S6 — Treatment effects on potential denitrification, soil CO2 emission rates, soil moisture and soil temperature (at 2 cm depth) year two after fire (n = 80×3 sampling dates – 15, 19 and 21 months after fire, except for soil moisture where n = 80×2 sampling dates – 19 and 21 months after fire). Treatments are burn (B), elevated CO2 (CO2), increased precipitation (W), and N supply (N). Significant responses are indicated in bold (α = 0.05). The overall effect of the burn treatment was calculated as: % effect = 100×[burned−unburned]/unburned (n = 32×3 in the burned plots, n = 48×3 in the unburned plots). The overall effects of the CO2, precipitation, and N treatments were calculated as: % effect = 100×[elevated−ambient]/ambient (n = 40×3 in the elevated and ambient plots). (DOC) [file pone.0020105.s006.doc]

**Table S6**. Treatment effects on potential denitrification, soil CO_2_ emission rates, soil moisture and soil temperature (at 2 cm depth) year two after fire (n = 80 x 3 sampling dates – 15, 19 and 21 months after fire, except for soil moisture where n = 80 x 2 sampling dates – 19 and 21 months after fire)

|  | **Potential denitrification** | | **Soil CO_2_ emission** | | **Soil moisture** | | **Soil temperature** | |
| --- | --- | --- | --- | --- | --- | --- | --- | --- |
| **Treatment** | % effect | p-value | % effect | p-value | % effect | p-value | % effect | p-value |
| **B** | 24 | 0.18 | **22** | **0.004** | **9** | **0.02** | 3 | 0.07 |
| **CO_2_** | -4 | 0.49 | 7 | 0.59 | 4 | 0.19 | -0.4 | 0.74 |
| **W** | 2 | 0.85 | **-19** | **0.0004** | 1 | 0.42 | -0.4 | 0.36 |
| **N** | **49** | **<0.0001** | 8 | 0.40 | 4 | 0.18 | **-2** | **0.0001** |
| **B x CO_2_** |  | 0.74 |  | **0.02** |  | 0.20 |  | **0.04** |
| **B x W** |  | 0.50 |  | 0.80 |  | 0.33 |  | 0.30 |
| **B x N** |  | **0.0002** |  | 0.80 |  | 0.41 |  | 0.65 |
| **CO_2_ x W** |  | 0.23 |  | 0.86 |  | 0.70 |  | 0.84 |
| **CO_2_ x N** |  | 0.97 |  | 0.18 |  | 0.36 |  | 0.77 |
| **W x N** |  | 0.23 |  | 0.99 |  | 0.58 |  | 0.74 |
| **B x CO_2_ x W** |  | 0.10 |  | 0.99 |  | 0.26 |  | 0.79 |
| **B x CO_2_ x N** |  | 0.90 |  | 0.44 |  | 0.37 |  | 0.29 |
| **B x W x N** |  | 0.22 |  | 0.75 |  | 0.60 |  | 0.87 |
| **CO_2_ x W x N** |  | 0.26 |  | 0.86 |  | 0.94 |  | 0.34 |
| **B x CO_2_ x W x N** |  | 0.45 |  | 0.34 |  | 0.48 |  | 0.30 |
|  |  |  |  |  |  |  |  |  |
| **Time** |  | **<0.0001** |  | **0.0002** |  | **<0.0001** |  | **<0.0001** |
| **Time x B** |  | **0.004** |  | **0.03** |  | 0.99 |  | **0.0007** |
| **Time x CO_2_** |  | 0.43 |  | **0.003** |  | 0.40 |  | 0.80 |
| **Time x W** |  | **<0.0001** |  | 0.60 |  | **0.03** |  | 0.91 |
| **Time x N** |  | **<0.0001** |  | 0.92 |  | **0.0003** |  | **0.0009** |
| **Time x B x CO_2_** |  | 0.39 |  | 0.47 |  | 0.75 |  | 0.97 |
| **Time x B x W** |  | 0.99 |  | 0.29 |  | 0.17 |  | 0.28 |
| **Time x B x N** |  | 0.70 |  | 0.50 |  | 0.20 |  | 0.86 |
| **Time x CO_2_ x W** |  | 0.21 |  | 0.33 |  | 0.49 |  | 0.90 |
| **Time x CO_2_ x N** |  | 0.48 |  | **0.05** |  | 0.83 |  | 0.33 |
| **Time x W x N** |  | 0.59 |  | 0.90 |  | 0.38 |  | 0.18 |
| **Time x B x CO_2_ x W** |  | 0.12 |  | 0.31 |  | 0.47 |  | 0.97 |
| **Time x B x CO_2_ x N** |  | 0.85 |  | 0.32 |  | 0.99 |  | 0.40 |
| **Time x B x W x N** |  | **0.009** |  | 0.27 |  | 0.20 |  | 0.20 |
| **Time x CO_2_ x W x N** |  | 0.42 |  | 0.49 |  | 0.47 |  | 0.72 |
| **Time x B x CO_2_ x W x N** |  | 0.77 |  | 0.92 |  | 0.62 |  | 0.50 |

Treatments are burn (B), elevated CO_2_ (CO_2_), increased precipitation (W), and N supply (N). Significant responses are indicated in bold (α = 0.05). The overall effect of the burn treatment was calculated as: % effect = 100 x [burned – unburned] / unburned (n = 32 x 3 in the burned plots, n = 48 x 3 in the unburned plots). The overall effects of the CO_2_, precipitation, and N treatments were calculated as: % effect = 100 x [elevated – ambient] / ambient (n = 40 x 3 in the elevated and ambient plots).
