# Supplementary material for: Global Change Could Amplify Fire Effects on Soil Greenhouse Gas Emissions
Source: PLoS One. 2011 Jun 8;6(6):e20105. doi: 10.1371/journal.pone.0020105 (PMC3110610; doi:10.1371/journal.pone.0020105)
Supplement: Table S7 — Treatment effects on potential denitrification, soil CO2 emission rates, soil moisture and soil temperature (at 2 cm depth) year three after fire (n = 80). Treatments are burn (B), elevated CO2 (CO2), increased precipitation (W), and N supply (N). Significant responses are indicated in bold (α = 0.05). The overall effect of the burn treatment was calculated as: % effect = 100×[burned−unburned]/unburned (n = 32 in the burned plots, n = 48 in the unburned plots). The overall effects of the CO2, precipitation, and N treatments were calculated as: % effect = 100×[elevated−ambient]/ambient (n = 40 in the elevated and ambient plots). (DOC) [file pone.0020105.s007.doc]

**Table S7**. Treatment effects on potential denitrification, soil CO_2_ emission rates, soil moisture and soil temperature (at 2 cm depth) year three after fire (n = 80)

|  | **Potential denitrification** | | **Soil CO_2_ emission** | | **Soil moisture** | | **Soil temperature** | |
| --- | --- | --- | --- | --- | --- | --- | --- | --- |
| **Treatment** | % effect | p-value | % effect | p-value | % effect | p-value | % effect | p-value |
| **B** | **63** | **0.003** | **41** | **0.009** | **12** | **0.004** | -1 | 0.62 |
| **CO_2_** | 19 | 0.44 | 5 | 0.73 | **14** | **0.009** | -3 | 0.46 |
| **W** | **27** | **0.04** | -8 | 0.31 | 2 | 0.60 | 0.3 | 0.91 |
| **N** | **39** | **0.0004** | -7 | 0.58 | 3 | 0.13 | **-4** | **<0.0001** |
| **B x CO_2_** |  | 0.27 |  | 0.71 |  | 0.14 |  | 0.06 |
| **B x W** |  | 0.47 |  | 0.12 |  | 0.12 |  | 0.58 |
| **B x N** |  | **0.03** |  | 0.58 |  | **0.005** |  | 0.72 |
| **CO_2_ x W** |  | **0.01** |  | 0.77 |  | 0.86 |  | 0.81 |
| **CO_2_ x N** |  | 0.72 |  | 0.53 |  | 0.43 |  | 0.73 |
| **W x N** |  | 0.48 |  | 0.65 |  | 0.61 |  | 0.48 |
| **B x CO_2_ x W** |  | 0.96 |  | 0.38 |  | 0.62 |  | 0.66 |
| **B x CO_2_ x N** |  | 0.32 |  | 0.93 |  | 0.19 |  | 0.61 |
| **B x W x N** |  | 0.56 |  | 0.62 |  | 0.95 |  | 0.62 |
| **CO_2_ x W x N** |  | 0.06 |  | 0.61 |  | 0.57 |  | 0.33 |
| **B x CO_2_ x W x N** |  | 0.82 |  | 0.08 |  | 0.50 |  | 0.07 |

Treatments are burn (B), elevated CO_2_ (CO_2_), increased precipitation (W), and N supply (N). Significant responses are indicated in bold (α = 0.05). The overall effect of the burn treatment was calculated as: % effect = 100 x [burned – unburned] / unburned (n = 32 in the burned plots, n = 48 in the unburned plots). The overall effects of the CO_2_, precipitation, and N treatments were calculated as: % effect = 100 x [elevated – ambient] / ambient (n = 40 in the elevated and ambient plots).
