# Supplementary material for: Global Change Could Amplify Fire Effects on Soil Greenhouse Gas Emissions
Source: PLoS One. 2011 Jun 8;6(6):e20105. doi: 10.1371/journal.pone.0020105 (PMC3110610; doi:10.1371/journal.pone.0020105)
Supplement: Table S8 — Treatment effects on gross N mineralization, gross nitrification and soil NH4 + and NO3 − concentrations year two after fire (n = 80×3 sampling dates – 15, 19 and 21 months after fire, except for N mineralization where n = 80×2 sampling dates – 19 and 21 months after fire). Treatments are burn (B), elevated CO2 (CO2), increased precipitation (W), and N supply (N). Significant responses are indicated in bold (α = 0.05). The overall effect of the burn treatment was calculated as: % effect = 100×[burned−unburned]/unburned (n = 32×3 in the burned plots, n = 48×3 in the unburned plots). The overall effects of the CO2, precipitation, and N treatments were calculated as: % effect = 100×[elevated−ambient]/ambient (n = 40×3 in the elevated and ambient plots). (DOC) [file pone.0020105.s008.doc]

**Table S8. Treatment effects on gross N mineralization, gross nitrification and soil NH_4_^+^ and NO_3_^-^ concentrations year two after fire (n = 80 x 3 sampling dates – 15, 19 and 21 months after fire, except for N mineralization where n = 80 x 2 sampling dates – 19 and 21 months after fire)**

|  | **Gross N mineralization** | | **Gross nitrification** | | **Soil NH_4_^+^ concentrations** | | **Soil NO_3_^-^ concentrations** | |
| --- | --- | --- | --- | --- | --- | --- | --- | --- |
| **Treatment** | % effect | p-value | % effect | p-value | % effect | p-value | % effect | p-value |
| **B** | -10 | 0.06 | -13 | 0.52 | **-23** | **0.03** | -31 | 0.16 |
| **CO_2_** | -3 | 0.09 | 8 | 0.65 | -8 | 0.09 | 70 | 0.12 |
| **W** | -8 | 0.09 | -8 | 0.91 | -5 | 0.42 | 6 | 0.84 |
| **N** | **28** | **0.0001** | **72** | **0.003** | **96** | **<0.0001** | **771** | **<0.0001** |
| **B x CO_2_** |  | 0.58 |  | 0.49 |  | **0.02** |  | 0.75 |
| **B x W** |  | 0.99 |  | 0.94 |  | 0.55 |  | 0.92 |
| **B x N** |  | 0.93 |  | 0.14 |  | 0.67 |  | 0.26 |
| **CO_2_ x W** |  | **0.004** |  | 0.24 |  | 0.81 |  | 0.35 |
| **CO_2_ x N** |  | 0.53 |  | 0.44 |  | 0.16 |  | 0.15 |
| **W x N** |  | 0.55 |  | 0.43 |  | 0.16 |  | 0.32 |
| **B x CO_2_ x W** |  | 0.95 |  | **0.04** |  | 0.16 |  | 0.28 |
| **B x CO_2_ x N** |  | 0.12 |  | 0.30 |  | 0.61 |  | 0.96 |
| **B x W x N** |  | 0.38 |  | 0.07 |  | 0.61 |  | 0.48 |
| **CO_2_ x W x N** |  | 0.30 |  | 0.59 |  | 0.82 |  | 0.94 |
| **B x CO_2_ x W x N** |  | 0.39 |  | 0.23 |  | 0.56 |  | 0.40 |
|  |  |  |  |  |  |  |  |  |
| **Time** |  | **0.006** |  | **<0.0001** |  | **<0.0001** |  | **0.0001** |
| **Time x B** |  | 0.88 |  | 0.07 |  | **0.003** |  | **0.01** |
| **Time x CO_2_** |  | **0.02** |  | 0.07 |  | 0.41 |  | 0.20 |
| **Time x W** |  | 0.65 |  | 0.51 |  | 0.53 |  | 0.76 |
| **Time x N** |  | 0.56 |  | **0.009** |  | **0.005** |  | **<0.0001** |
| **Time x B x CO_2_** |  | 0.79 |  | 0.51 |  | 0.07 |  | 0.79 |
| **Time x B x W** |  | 0.80 |  | 0.81 |  | 0.52 |  | 0.94 |
| **Time x B x N** |  | 0.94 |  | 0.29 |  | 0.17 |  | **0.0006** |
| **Time x CO_2_ x W** |  | 0.69 |  | 0.17 |  | 0.25 |  | 0.19 |
| **Time x CO_2_ x N** |  | 0.49 |  | 0.70 |  | 0.67 |  | 0.34 |
| **Time x W x N** |  | 0.07 |  | 0.74 |  | 0.50 |  | 0.67 |
| **Time x B x CO_2_ x W** |  | 0.10 |  | 0.92 |  | 0.38 |  | 0.68 |
| **Time x B x CO_2_ x N** |  | 0.67 |  | 0.15 |  | 0.44 |  | 0.45 |
| **Time x B x W x N** |  | 0.80 |  | 0.21 |  | 0.70 |  | 0.38 |
| **Time x CO_2_ x W x N** |  | 0.15 |  | 0.41 |  | 0.19 |  | 0.19 |
| **Time x B x CO_2_ x W x N** |  | 0.59 |  | 0.53 |  | 0.11 |  | 0.60 |

Treatments are burn (B), elevated CO_2_ (CO_2_), increased precipitation (W), and N supply (N). Significant responses are indicated in bold (α = 0.05). The overall effect of the burn treatment was calculated as: % effect = 100 x [burned – unburned] / unburned (n = 32 x 3 in the burned plots, n = 48 x 3 in the unburned plots). The overall effects of the CO_2_, precipitation, and N treatments were calculated as: % effect = 100 x [elevated – ambient] / ambient (n = 40 x 3 in the elevated and ambient plots).
