# Supplementary material for: Global Change Could Amplify Fire Effects on Soil Greenhouse Gas Emissions
Source: PLoS One. 2011 Jun 8;6(6):e20105. doi: 10.1371/journal.pone.0020105 (PMC3110610; doi:10.1371/journal.pone.0020105)
Supplement: Table S9 — Treatment effects on potential ammonia and nitrite oxidation year two after fire (n = 80×2 sampling dates – 19 and 21 months after fire). Treatments are burn (B), elevated CO2 (CO2), increased precipitation (W), and N supply (N). Significant responses are indicated in bold (α = 0.05). The overall effect of the burn treatment was calculated as: % effect = 100×[burned−unburned]/unburned (n = 32×2 in the burned plots, n = 48×2 in the unburned plots). The overall effects of the CO2, precipitation, and N treatments were calculated as: % effect = 100×[elevated−ambient]/ambient (n = 40×2 in the elevated and ambient plots). (DOC) [file pone.0020105.s009.doc]

**Table S9.** Treatment effects on potential ammonia and nitrite oxidation year two after fire (n = 80 x 2 sampling dates – 19 and 21 months after fire)

|  | **Potential**  **ammonia oxidation** | | **Potential**  **nitrite oxidation** | |
| --- | --- | --- | --- | --- |
| **Treatment** | % effect | p-value | % effect | p-value |
| **B** | -10 | 0.59 | -12 | 0.47 |
| **CO_2_** | 9 | 0.44 | 12 | 0.48 |
| **W** | 15 | 0.10 | **-17** | **0.003** |
| **N** | **50** | **<0.0001** | -4 | 0.86 |
| **B x CO_2_** |  | 0.91 |  | **0.03** |
| **B x W** |  | 0.70 |  | 0.38 |
| **B x N** |  | 0.16 |  | 0.15 |
| **CO_2_ x W** |  | 0.47 |  | 0.26 |
| **CO_2_ x N** |  | 0.71 |  | 0.18 |
| **W x N** |  | 0.47 |  | 0.52 |
| **B x CO_2_ x W** |  | 0.24 |  | 0.61 |
| **B x CO_2_ x N** |  | 0.57 |  | 0.39 |
| **B x W x N** |  | 0.50 |  | **0.009** |
| **CO_2_ x W x N** |  | 0.73 |  | 0.11 |
| **B x CO_2_ x W x N** |  | 0.94 |  | 0.78 |
|  |  |  |  |  |
| **Time** |  | 0.29 |  | 0.61 |
| **Time x B** |  | 0.67 |  | **0.03** |
| **Time x CO_2_** |  | 0.44 |  | 0.27 |
| **Time x W** |  | 0.80 |  | 0.75 |
| **Time x N** |  | 0.60 |  | 0.13 |
| **Time x B x CO_2_** |  | 0.79 |  | 0.92 |
| **Time x B x W** |  | 0.30 |  | 0.16 |
| **Time x B x N** |  | **0.004** |  | 0.12 |
| **Time x CO_2_ x W** |  | 0.53 |  | 0.19 |
| **Time x CO_2_ x N** |  | 0.94 |  | 0.10 |
| **Time x W x N** |  | 0.82 |  | 0.99 |
| **Time x B x CO_2_ x W** |  | 0.23 |  | 0.51 |
| **Time x B x CO_2_ x N** |  | 0.14 |  | 0.81 |
| **Time x B x W x N** |  | 0.48 |  | 0.23 |
| **Time x CO_2_ x W x N** |  | 0.54 |  | 0.78 |
| **Time x B x CO_2_ x W x N** |  | 0.69 |  | 0.19 |

Treatments are burn (B), elevated CO_2_ (CO_2_), increased precipitation (W), and N supply (N). Significant responses are indicated in bold (α = 0.05). The overall effect of the burn treatment was calculated as: % effect = 100 x [burned – unburned] / unburned (n = 32 x 2 in the burned plots, n = 48 x 2 in the unburned plots). The overall effects of the CO_2_, precipitation, and N treatments were calculated as: % effect = 100 x [elevated – ambient] / ambient (n = 40 x 2 in the elevated and ambient plots).
